# Integrated Care for Older Adults Improves Perceived Quality of Care: Results of a Randomized Controlled Trial of Embrace

**DOI:** 10.1007/s11606-016-3742-y

**Published:** 2016-06-06

**Authors:** Ronald J. Uittenbroek, Hubertus P. H. Kremer, Sophie L. W. Spoorenberg, Sijmen A. Reijneveld, Klaske Wynia

**Affiliations:** 1 Department of Health Sciences, Community and Occupational Medicine, University Medical Center Groningen, University of Groningen, P.O. BOX 196, 9700 AD Groningen, The Netherlands; 2 Department of Neurology, University Medical Center Groningen, University of Groningen, P.O. BOX 30001, 9700 RB Groningen, The Netherlands

**Keywords:** integrated care, patient-centered care, older adults, quality of care, randomized controlled trial

## Abstract

**Background:**

All community-living older adults might benefit from integrated care, but evidence is lacking on the effectiveness of such services for perceived quality of care.

**Objective:**

To examine the impact of Embrace, a community-based integrated primary care service, on perceived quality of care.

**Design:**

Stratified randomized controlled trial.

**Participants:**

Integrated care and support according to the “Embrace” model was provided by 15 general practitioners in the Netherlands. Based on self-reported levels of case complexity and frailty, a total of 1456 community-living older adults were stratified into non-disease-specific risk profiles (“Robust,” “Frail,” and “Complex care needs”), and randomized to Embrace or control groups.

**Intervention:**

Embrace provides integrated, person-centered primary care and support to all older adults living in the community, with intensity of care dependent on risk profile.

**Measurements:**

Primary outcome was quality of care as reported by older adults on the Patient Assessment of Integrated Elderly Care (PAIEC). Effects were assessed using mixed model techniques for the total sample and per risk profile. Professionals’ perceived level of implementation of integrated care was evaluated within the Embrace condition using the Assessment of Integrated Elderly Care.

**Key results:**

Older adults in the Embrace group reported a higher level of perceived quality of care than those in the control group (B = 0.33, 95 % CI = 0.15-0.51, ES *d* = 0.19). The advantages of Embrace were most evident in the “Frail” and “Complex care needs” risk profiles. We found no significant advantages for the “Robust” risk profile. Participating professionals reported a significant increase in the perceived level of implementation of integrated care (ES *r* = 0.71).

**Conclusions:**

This study shows that providing a population-based integrated care service to community-living older adults improved the quality of care as perceived by older adults and participating professionals.

**Electronic supplementary material:**

The online version of this article (doi:10.1007/s11606-016-3742-y) contains supplementary material, which is available to authorized users.

## BACKGROUND

One of the main challenges for today’s healthcare systems is organizing high-quality, comprehensive, person-centered, integrated care and support for older adults.^1^^–^ 3 Addressing these challenges requires comprehensive, and thus complex, interventions in community-based healthcare systems.^4^^–^ 6 However, evidence is still lacking regarding the effectiveness of such interventions on patient outcomes and quality of care as perceived by older adults.^7^^,^ 8

A promising approach to delivering age-specific long-term care and support lies in integrated care services. The Chronic Care Model (CCM)^9^ is a well-known framework that can be used to develop such services. The CCM integrates community social care and healthcare services, and has four interdependent key elements: self-management support, delivery system design, decision support, and clinical information systems. An example of a CCM-based integrated care service is the “Guided Care" model for multimorbid older adults.^10^ Within Guided Care, a physician–nurse primary care team provides care for the most complex patients, with the aim of increasing both quality of care and quality of life while lowering costs. Studies on this service have shown encouraging effects on perceptions of quality of care among older adults.^11^ However, as is typical of such initiatives, this service targets only those already in need of care. It is well known that the health status of older adults can change quickly, taking a sudden turn for the worse.^4^ Moreover, preventive and proactive care may help to delay declining health status among older adults.^12^^–^ 16

Therefore, integrated care models could be even more effective if they targeted the general population of older adults. On the other hand, delivering the same care intensity to all older adults is neither needed nor sustainable.^17^ Thus a more practical solution may be in combining integrated care models with population health management models. These models can help stratify a population of community-living older adults into risk profiles with corresponding levels of care and support that are non-disease- and non-service-specific.^18^

Embrace (in Dutch: “SamenOud”)^19^ is an integrated care service designed for all community-living older adults, which combines the CCM with risk profiles based on a population health management model, the Kaiser Permanente Triangle.^20^ In the Embrace model, participating older adults are stratified into the risk profiles based on self-reported levels of “case complexity”^21^ and level of “frailty,”^22^ and care and support are then offered by a multidisciplinary Elderly Care Team, with intensity determined by individual risk profiles. Embrace aims to integrate health and social services with preventive care and support, and was developed to improve patient health outcomes as well as quality of care, service use, and cost.

The aim of the present study was to assess the effects of Embrace on the quality of care as perceived by community-living older adults and participating professionals.

## METHODS

### Design

We performed a randomized controlled trial (RCT) from January 2012 through March 2013, in which older adult participants were stratified into risk profiles and equally allocated to intervention and control groups, as reported previously.^19^ Alongside the RCT, the perceived “level of implementation of integrated elderly care” of participating professionals was evaluated within the intervention arm. The Medical Ethical Committee of the University Medical Center Groningen assessed our study proposal and concluded that their approval was not required (Reference METc2011.108). The study was performed in accordance with the tenets of the Declaration of Helsinki.^23^

### Sample Size

We calculated the sample size for participants based on a clinically relevant change in the self-reported health status visual analogue scale as measured with the visual analogue scale of the EuroQol-5D (EQ-5D-VAS).^24^ We needed 1062 older adults to detect a six-point difference on the EQ-5D-VAS with a standard deviation of 14 points, power of 80 %, and two-sided *p* value of 0.05 in the smallest risk profile (“Frail”). With an estimated loss to follow-up of 30 % and 30 % non-response, 2178 older adults needed to be invited. With an average of 200 enlisted older adults per general practitioner (GP) practice (also referred to as family physicians or primary care physicians), 11 GP practices were required.

### Participants and Procedure

First, we invited all 24 GPs in three municipalities in the province of Groningen, the Netherlands, to participate. After 15 GPs consented to participate, we stopped recruitment. Participating GPs were evenly distributed over the three municipalities. This included six GPs who were working as solo GPs and nine GPs who were part of a partnership or group practice. This distribution of practice types is comparable to that in the rest of the Netherlands.^25^ Next, we invited all adults aged 75 years and over from these 15 GP practices. Exclusion criteria were long-term admission to a nursing home (the equivalent of a US skilled nursing facility), involvement in a comparable integrated care service, or participation in another scientific study.

Eligible older adults received a letter from their GP with general information about the study. Once their written consent was provided, these adults completed self-report questionnaires and answered questions regarding demographic and health-related characteristics, both at baseline and after 12 months of follow-up. Participating professionals were asked to complete a questionnaire both at baseline and 12-month follow-up.

### Stratified Randomization and Blinding

Participating older adults were stratified into three risk profiles based on their responses on the questionnaires regarding case complexity (INTERMED for the Elderly, self-assessment, INTERMED-E-SA)^21^ and frailty (Groningen Frailty Indicator, GFI).^22^ The risk profiles were as follows: “Robust” (INTERMED-E-SA score < 16 and GFI score < 5), “Frail” (INTERMED-E-SA score < 16 and GFI score ≥ 5), and “Complex care needs” (INTERMED-E-SA score ≥ 16, irrespective of GFI score). After stratification, we performed a concealed and computerized balanced allocation procedure to achieve equal distribution between the intervention and control groups with regard to participants’ demographic and health-related characteristics.

Due to the study design, Elderly Care Team members knew which participants were assigned to Embrace. The participating older adults were informed in writing as to whether they were assigned to an intervention or a control group. For practical reasons, the data manager was not blinded; researchers, however, were masked until analysis.

### Intervention

For Embrace, a GP-led Elderly Care Team was assembled within each participating GP practice, in which the GP remained responsible for writing prescriptions and implementing the interventions. The Elderly Care Team additionally consisted of an elderly care physician (i.e., a nursing home physician), a community nurse, and a social worker. All Elderly Care Team members completed a training program. The initial training for the GPs (3 days in total) focused on team and population management and on essential themes such as multimorbidity and polypharmacy. Social workers and district nurses received specific training in areas such as case management and shared decision making during an 8-day initial training program. All members of the Elderly Care Teams received monthly on-the-job coaching during their team meetings.

The Elderly Care Team provided older adults with comprehensive, patient-centered, proactive, and preventive care and support. Members met monthly for consultation at the participating GP practice. The intensity and focus of care and support at the patient level differed by risk profile in terms of number of contacts, main focus, health-related vs. social problems, and individual vs. group approach. Older adults within the “Frail” and “Complex care needs” profiles received individual care and support from a case manager, a social worker, and community nurse, respectively. They visited these older adults at home once or twice a month, and focused specifically on problems experienced by older adults, i.e., emotional and exercise tolerance functions.^26^

Elderly Care Team monitoring of older adults within the “Robust” profile consisted in reviewing their medical files, medications, and self-reported levels of frailty and case complexity once a year. Participating “Robust” older adults were encouraged to contact the Elderly Care Team if their health or life situation changed. They received a questionnaire on changes in their health or life situation and directions for follow-up. The Elderly Care Team acted proactively in cases of suspected deterioration in health status (e.g., increased forgetfulness, sudden loss of weight) or an imminent loss of support from the informal network (e.g., an overburdened caregiver) for older adults who received case management.

Finally, all participating older adults were offered a self-management support and prevention program that included, for example, community meetings and newsletters emphasizing the need for prevention and healthy lifestyles while maintaining self-management abilities. See Online Supplementary Table S1 for additional details on intensity, duration, and cost per risk profile.

### Care as Usual

The control group received usual care as provided by their GP and the local health and social care organizations. In the Netherlands, municipalities are responsible for social care and health promotion, which is government (tax)-funded. Basic healthcare insurance is obligatory, and covers almost all primary and secondary healthcare. The GP acts as gatekeeper for specialized medical care. GP visit rates increases with age, from four visits per year at ages 45–64, to ten visits annually at ages 75 years and older.^27^

### Primary Outcome

The primary outcome for this study was quality of care as reported by older adults on the Patient Assessment of Integrated Elderly Care (PAIEC) scale (see Online Appendix).^28^ The PAIEC is a modified version of the Patient Assessment of Chronic Illness Care (PACIC),29 and consists of 20 items divided into three subscales: “Patient activation and contextual information,” “Goal-setting and problem-solving,” and “Coordination and follow-up.” Each item was scored on a five-point scale ranging from 1 (never) to 5 (always), or the response option “does not apply.” The response option “does not apply” and missing values were recoded to “never” to gain a more realistic estimate of the integrated care received and its intensity. Next, we calculated index scores by subtracting the minimum scale score from the raw scale score, dividing this by the scale score range, and then multiplying by 100, resulting in scores ranging from 0 to 100, with higher scores reflecting better perceived quality of care. We then normalized the skewed PAIEC distribution by a power (square root) transformation (√[PAIEC index scores +1]).^30^^,^ 31

### Secondary Outcome

The secondary outcome for this study was the “level of implementation of integrated elderly care” as reported by the professionals on the Assessment of Integrated Elderly Care (AIEC) scale. The AIEC is a modified version of the validated Assessment of Chronic Illness Care (ACIC version 3.5), which assesses whether the care and support provided is in accordance with the Chronic Care Model.^32^^,^ 33 The AIEC consists of 34 items divided into 7 subscales: “Healthcare organization,” “Community linkages,” “Self-management support,” “Delivery system design,” “Decision support,” “Clinical information systems,” and “Integration of CCM elements.” Each item was scored on a scale of 0 to 11, and total and subscale sum scores were calculated after recoding missing items into score “0,” and converted into index scores (0–100), with higher scores reflecting a higher “level of implementation of integrated elderly care.” This questionnaire was translated following a forward–backward procedure,34 and then modified for the care offered–in other words, “chronic care” was converted into “care and support for older adults.”

### Analysis

We first constructed a flow diagram and described baseline characteristics of participating older adults at the overall sample level and per risk profile. We tested differences between groups using the *t* test for continuous variables, the Mann–Whitney *U* test for ordinal and not normally distributed continuous variables, and the Chi-square test for categorical variables.

We next assessed the effects of Embrace on quality of care in terms of regression coefficients (B) with 95 % confidence intervals (CI), using multi-level analyses, with older adults as lower level and GP practices as higher level, adjusted for age and gender. We used an intention-to-treat (ITT) analysis, with the last observation carried forward, followed by a complete case analysis–both at the total sample and risk profile levels. In addition, we assessed effect sizes by calculating Cohen’s *d* for regression coefficients, considering an effect size ≥ 0.2 to be clinically relevant.^35^^,^ 36

Finally, we assessed changes in the level of implementation of integrated elderly care from the perspective of the Elderly Care Team members. We combined the team members’ scores with the aggregated total and subscale scores, and then assessed differences between pre-test and post-test scores using the Wilcoxon signed-rank test. We assessed effect sizes by calculating the nonparametric effect size *r,* considering an effect size ≥ 0.1 to be clinically relevant.^36^^,^ 37 We conducted all statistical analyses using SPSS (IBM SPSS Statistics for Windows, version 22, 2013; IBM Corp., Armonk, NY, USA).

## RESULTS

**Figure 1:**
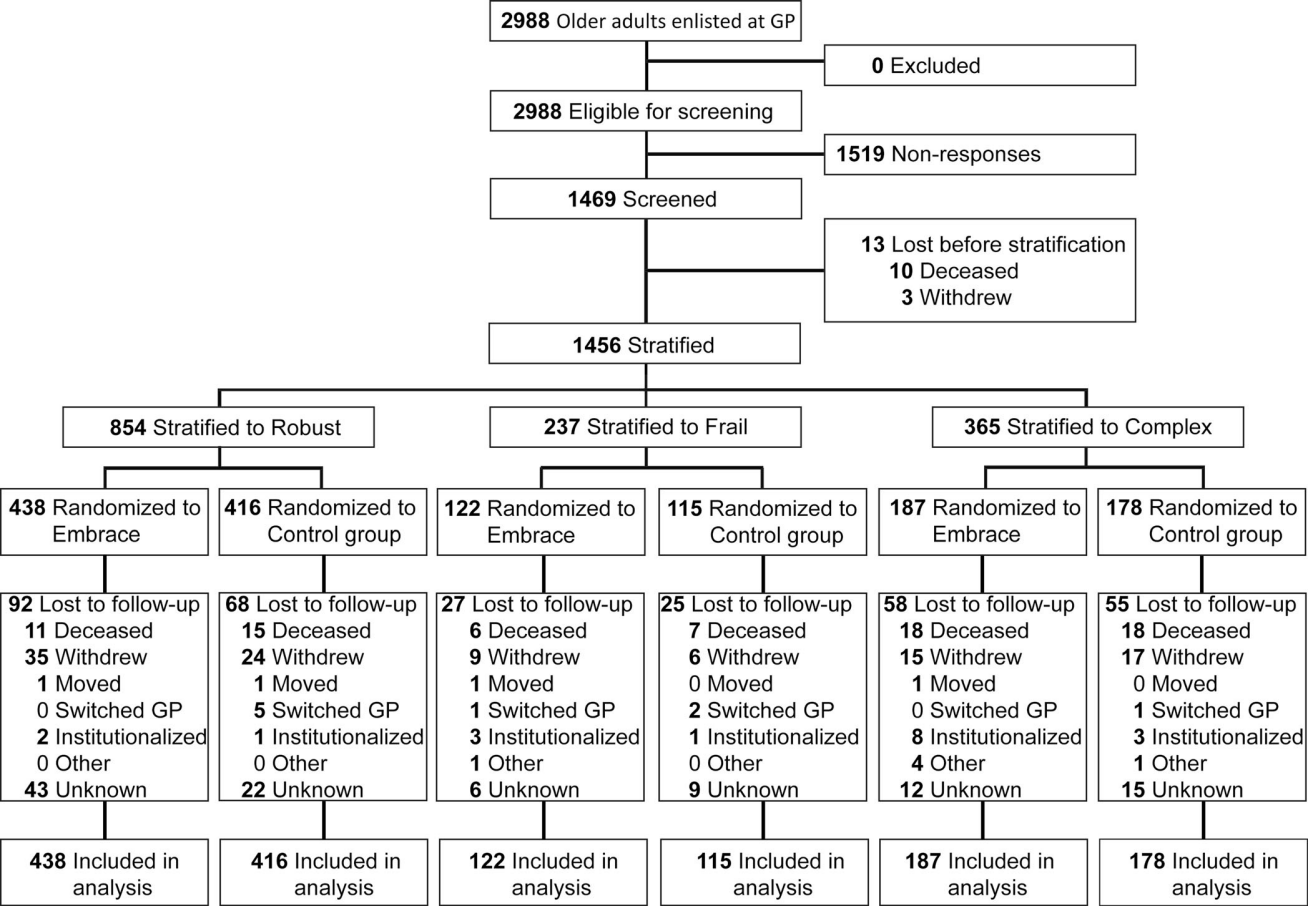
Flow of participants in the Embrace study**.**

Figure 1 shows the flow of older adults through the study. In total, 1456 of the 2988 eligible older adults were included in the ITT analyses. Women, the oldest older adults, and older adults who lived in rural areas declined participation more frequently (all *p* < 0.01). We found no between-group differences in loss to follow-up for either the total sample or risk profiles. Older adults lost to follow-up (*n* = 325, 22 %) were significantly (*p* < 0.01) older and frailer, had more complex care needs and a lower health status, and received home care more often.

### Baseline Characteristics

Table 1 shows the baseline characteristics of the 1456 participating older adults. We found no statistically significant differences between the Embrace and control groups, with the exception of “home help received during the past year” in the risk profile “Complex care needs” (*p* = 0.04).

**Table 1:** Characteristics of Participating Older Adults at Baseline

Baseline characteristic	Total sample		Complex care needs		Frail		Robust	
	(*n* = 1456)		(*n* = 365)		(*n* = 237)		(*n* = 854)	
	Embrace	Control group	Embrace	Control group	Embrace	Control group	Embrace	Control group
Number of participants	747	709	187	178	122	115	438	416
Age, mean (SD)	80.7 (4.5)	80.8 (4.7)	81.8 (4.6)	81.5 (4.9)	81.6 (5.1)	82.8 (5.5)	79.9 (4.0)	79.9 (4.1)
Female, *n* (%)	405 (54.2)	394 (55.6)	121 (64.7)	115 (64.6)	82 (67.2)	80 (69.6)	202 (46.1)	199 (47.8)
Marital status, *n* (%)								
Married/unmarried or living together	427 (57.2)	417 (59.0)	100 (53.5)	99 (55.6)	45 (36.9)	42 (36.8)	282 (64.4)	276 (66.5)
Widow(er), single or divorced	320 (42.8)	290 (41.0)	87 (46.5)	79 (44.4)	77 (63.1)	72 (63.2)	156 (35.6)	139 (33.5)
Living situation, *n* (%)								
Community-living with others	435 (57.6)	407 (57.1)	101 (54.0)	95 (53.4)	49 (40.3)	36 (31.3)	279 (63.7)	276 (66.3)
Community-living single	302 (40.0)	279 (39.1)	75 (40.1)	70 (39.3)	71 (58.2)	69 (60.0)	154 (35.2)	137 (32.9)
Residential care	14 (1.9)	23 (3.2)	10 (5.4)	12 (6.7)	2 (1.6)	9 (7.8)	2 (0.5)	2 (0.5)
Low educational level, *n* (%)	370 (49.9)	374 (53.4)	106 (57.0)	116 (66.3)	66 (54.1)	69 (60.0)	198 (45.7)	189 (46.0)
Low household income, *n* (%)	261 (35.7)	231 (33.6)	80 (43.5)	77 (43.8)	53 (44.5)	51 (45.1)	128 (29.9)	103 (25.9)
Chronic conditions, median (IQR)	2 (1-3)	2 (1-3)	3 (2-5)	3 (2-5)	3 (1-4)	3 (2-4)	1 (1-2)	1 (1-2)
Hospitalized, *n* (%)	138 (19.0)	123 (17.5)	63 (35.0)	49 (27.5)	17 (14.0)	19 (16.7)	58 (13.6)	55 (13.4)
Received home care, *n* (%)	89 (12.1)	69 (9.8)	47 (26.4)	42 (23.9)	24 (20.0)	14 (12.4)	18 (4.1)	13 (3.2)
Received home help, *n* (%)	211 (28.4)	194 (27.6)	105 (57.4) *	82 (46.3) *	48 (39.3)	51 (44.7)	58 (13.3)	61 (14.8)
Complexity of care needs								
IM-E-SA, median (IQR)	10 (6-15)	10 (6-15)	19 (17-22)	19.5 (17-24)	12 (10-14)	12 (9-13)	7 (5-10)	8 (5-10)
Frailty								
GFI, median (IQR)	3 (2-6)	3 (2-6)	7 (5-8)	7 (5-9)	6 (5-7)	6 (5-7)	2 (1-3)	2 (1-3)
Health Status								
EQ-5D-3 L, means (SD)	0.79 (0.15)	0.78 (0.16)	0.65 (0.16)	0.64 (0.17)	0.74 (0.11)	0.74 (0.13)	0.86 (0.10)	0.86 (0.09)
EQ-5D-VAS, means (SD)	70.7 (17.5)	69.8 (18.3)	56.7 (16.7)	53.8 (19.4)	67.2 (15.6)	70.0 (13.5)	77.7 (14.1)	76.5 (14.5)

SD = standard deviation, IQR = interquartile range

Low education: primary school, low vocational training, or less

Low household income:eew < €1351 per month

IM-E-SA = Intermed Elderly Self-assessment, GFI = Groningen Frailty Indicator, EQ-5D = EuroQol health-related quality of life, VAS = visual analogue scale

* Indicates significant differences between groups (*p* = 0.04)

### Primary Outcome: Perceived Quality of Care

Table 2 shows the improvement in quality of care reported by older adults, based on the ITT analyses. Overall, older adults in the Embrace group perceived greater improvement in quality of care as reflected on the PAIEC than those in the control group (B = 0.33, 95 % CI = 0.15-0.51, ES *d* = 0.19). Effect sizes (*d*) were trivial to small. Embrace yielded the most improvement for the “Frail” and “Complex care needs” risk profiles, and no significant advantages for the “Robust” risk profile. For the risk profile “Frail,” all PAIEC scores differed significantly and indicated clinically relevant differences (small to medium effect sizes). PAIEC scores for the risk profile “Complex care needs” differed significantly for the total scale score and the subscale “Activation,” with both indicating clinically relevant differences (small effect sizes), while no significant advantages were found for the subscales “Goal-setting and problem-solving” and “Coordination and follow-up.” Complete case analyses confirmed ITT findings (not shown). Differences between GP practices were trivial.

**Table 2:** Outcomes Regarding Older Adults’ Reported Quality of Care on the PAIEC* Scores Based on Intention-to-Treat Analyses (*n* = 1456)

PAIEC	Embrace			Control group			Difference in improvement T1 vs. T0 between Embrace and control group		
	T0 (Baseline) Mean (SD)	T1 (Follow-up) Mean (SD)	Change Mean (SD)	T0 (Baseline) Mean (SD)	T1 (Follow-up) Mean (SD)	Change Mean (SD)	B (95 % CI)	*p*-value	ES
Total sample	*n* = 747			*n* = 709					
Total score	2.32 (1.91)	2.65 (2.14)	0.30 (2.08)	2.32 (1.82)	2.32 (1.89)	−0.02 (2.03)	0.33 (0.15;0.51)	<0.001	0.19
Activation	2.52 (2.24)	2.81 (2.42)	0.32 (1.92)	2.54 (2.16)	2.52 (2.15)	0.05 (1.90)	0.32 (0.11;0.53)	0.003	0.16
Goal	2.05 (1.96)	2.37 (2.22)	0.38 (1.93)	2.04 (1.91)	2.09 (2.03)	0.00 (1.77)	0.28 (0.08;0.47)	0.006	0.14
Coordination	1.99 (1.89)	2.37 (2.16)	0.33 (1.75)	1.95 (1.76)	1.95 (1.81)	0.00 (1.69)	0.38 (0.19;0.57)	<0.001	0.21
Complex care needs	*n* = 187			*n* = 178					
Total score	3.52 (2.24)	3.94 (2.34)	0.36 (2.62)	3.48 (2.06)	3.45 (2.34)	−0.19 (2.28)	0.44 (0.01;0.87)	0.044	0.21
Activation	3.87 (2.65)	4.22 (2.74)	0.57 (2.45)	3.93 (2.38)	3.74 (2.58)	0.09 (2.19)	0.54 (0.04;1.05)	0.035	0.22
Goal	2.95 (2.43)	3.52 (2.55)	0.39 (2.38)	3.07 (2.30)	3.16 (2.60)	0.14 (2.07)	0.48 (-0.00;0.96)	0.050	0.21
Coordination	3.07 (2.41)	3.46 (2.53)	0.42 (2.21)	2.80 (2.19)	2.94 (2.38)	−0.02 (1.94)	0.23 (-0.22;0.69)	0.312	0.11
Frail	*n* = 122			*n* = 115					
Total score	2.75 (2.19)	3.55 (2.41)	0.69 (2.14)	2.64 (1.90)	2.55 (1.86)	−0.06 (2.20)	0.89 (0.42;1.37)	<0.001	0.48
Activation	2.99 (2.40)	3.68 (2.62)	0.56 (2.26)	2.87 (2.28)	2.81 (2.20)	0.04 (2.03)	0.77 (0.21;1.32)	0.007	0.35
Goal	2.58 (2.41)	3.13 (2.68)	1.15 (2.54)	2.17 (1.99)	2.21 (2.03)	−0.22 (1.89)	0.56 (0.01;1.11)	0.045	0.26
Coordination	2.23 (2.14)	3.38 (2.54)	0.80 (2.02)	2.32 (2.02)	2.10 (1.87)	−0.09 (1.66)	1.32 (0.75;1.90)	<0.001	0.59
Robust	*n* = 438			*n* = 416					
Total score	1.69 (1.31)	1.85 (1.51)	0.17 (1.76)	1.73 (1.38)	1.77 (1.39)	0.06 (1.85)	0.13 (-0.07;0.33)	0.213	0.09
Activation	1.81 (1.62)	1.97 (1.77)	0.15 (1.51)	1.86 (1.66)	1.92 (1.65)	0.04 (1.73)	0.11 (-0.13;0.35)	0.378	0.06
Goal	1.51 (1.31)	1.67 (1.55)	0.17 (1.40)	1.57 (1.48)	1.60 (1.51)	0.00 (1.60)	0.12 (-0.10;0.33)	0.298	0.07
Coordination	1.46 (1.26)	1.63 (1.46)	0.16 (1.39)	1.48 (1.24)	1.48 (1.24)	0.03 (1.58)	0.16 (-0.04;0.36)	0.113	0.11

*Transformed scores are the square root of index scores

PAIEC = Patient Assessment of Integrated Elderly Care, B = unstandardized regression coefficient, CI = confidence interval

ES = effect size *d*; thresholds: < 0.2 trivial, ≥ 0.2–0.5 small, ≥ 0.5–0.8 medium, ≥ 0.8 large

### Secondary Outcome: Perceived Level of Implementation of Integrated Elderly Care

The 30 professionals, 12 of the 14 general practitioners, both elderly care physicians, 8 of the 9 district nurses, and 4 of the 5 social workers completed 49 (out of 56: 78.5 % response rate) AIEC questionnaires at baseline and follow-up. They reported that the average “level of implementation of integrated elderly care” at baseline was “basic” and, after 12 months, “reasonably good,” indicating clinically relevant improvement (Table 3).

**Table 3:** Outcomes Regarding Professionals’ Reported Level of Implementation on the AIEC (*n* = 30)

	T0 (Baseline)	T1 (Follow-up)	Change	Improvement T1 – T0		
AIEC	Mean (SD)	Mean (SD)	Mean (SD)	Z^*^	*p*-value	ES
Total score	46.5 (15.6)	57.4 (9.6)	10.8 (12.9)	−4.97	<0.001	0.71
Organization	51.3 (16.4)	57.9 (9.7)	6.6 (14.7)	−2.71	0.007	0.39
Community	46.6 (17.7)	55.6 (14.2)	8.9 (19.5)	−2.95	0.003	0.42
Self-management	42.5 (17.7)	55.4 (13.4)	12.9 (17.0)	−4.48	<0.001	0.64
Decision	40.8 (17.7)	48.1 (13.0)	7.2 (17.5)	−2.86	0.004	0.41
Delivery	51.9 (17.7)	63.1 (10.9)	11.2 (17.3)	−4.17	<0.001	0.60
Information systems	44.5 (16.4)	57.1 (12.9)	12.6 (17.5)	−4.41	<0.001	0.63
Integration	44.5 (21.0)	59.8 (13.8)	15.3 (20.4)	−4.34	<0.001	0.62

AIEC = Assessment of Integrated Elderly Care; scores: 0–24, limited support for integrated elderly care; 25–49, basic support for integrated elderly care; 50–74, reasonably good support for integrated elderly care; ≥75, fully developed integrated elderly care

*Wilcoxon signed-rank test

ES = effect size *r*; thresholds: < 0.1 trivial, ≥ 0.1–0.3 small, ≥ 0.3–0.5 medium, ≥ 0.5 large

## DISCUSSION

Our study results show that integrated care following the Embrace model improved the quality of care as perceived by community-living older adults and participating professionals. Advantages in perceived quality of integrated care were most evident for older adults receiving case management and were most prominent for older adults in the risk profile “Frail.” This may imply a positive effect from integrated care for older adults even when they are only “at risk” for poor health outcomes^22^ or for increasing case complexity,^21^ with no immediate need for professional care. Therefore, offering integrated care services should be considered for all older adults, particularly considering that proactive preventive care and support could be increasingly effective for their health outcomes in the longer run.^12^^,^ 14 ^,^ 16

We found no significant advantages from Embrace for “Robust” older adults, or for either “Goal-setting and problem-solving” or “Coordination and follow-up” for the risk profile “Complex care needs.” For these risk profiles, the differences between the Embrace and control groups may have been too small to detect. Older adults in the “Robust” profile of the Embrace group were offered a relatively low-intensity level of care and support, that is, a “self-management support and prevention” program, including community meetings, which were attended by about 25 % of these older adults. Furthermore, older adults were asked to evaluate the quality of care they received from all professionals, including those who were not part of Embrace. This may have diluted the effects of Embrace.

Elderly Care Team members reported that the “levels of support for integrated elderly care” improved from “basic” to “reasonably good” after 12 months. This may be due to the intensive training and coaching that they received before and during the intervention period.^38^ However, it also indicates room for improvement towards a goal of “fully developed integrated elderly care.”

Strengths of this study include its rigorous design, i.e., a randomized controlled trial with balanced allocation,^39^ and its large community-based sample. Another strength was the stratification of the population into risk profiles^40^ using two non-disease or service-specific identifiers (frailty and case complexity),^17^^,^ 18 which takes into account the aims of person-centered and integrated care.^3^ Although new and still in the process of validation,^41^ the measurement instruments used were the best available for classifying older adults into non-disease-specific risk profiles^18^ and for examining the quality of care and level of implementation of integrated care from the complementary perspectives of both older adults and professionals.^42^^,^ 43

Some (potential) limitations need to be addressed as well. First, given the differences between participants and non-participants at a 49 % participation rate, generalization of our findings requires further investigation. Second, we randomized participating older adults within the GP practices. This may have led to some contamination of the control group via the members of the Elderly Care Team, who received extensive training and were unblinded, causing some underestimation of the effects of Embrace. Third, older adults in the control groups were not blinded, which might have led to response bias. Finally, we had no control group for the professionals. Participating professionals may have responded in socially desirable ways, leading to some overestimation of the effects.

## Conclusions

Embrace is one of the first services with the aim of providing person-centered and integrated care to all community-living older adults. It was built on previous research regarding preventive and proactive care, and combines the well-known Chronic Care Model and the Kaiser Permanente Triangle. Our study showed that such a service can have positive effects on perceived quality of care. The effects we found were small to medium, and were most evident for older adults receiving case management. These results may have implications for policy, practice, and research.

First, our findings could support further development, integration, and funding of integrated care services for all older adults. Given the pivotal role of the Elderly Care Team, Embrace will be of particular value in healthcare systems that aim to strengthen primary care. Moreover, our findings can aid the further development of models such as patient-centered medical homes.^44^ Second, our findings need to be confirmed in other settings to assess their generalizability.^45^ Third, such evaluation could comprise other, more technical aspects of quality of care, including whether specific care processes were performed (e.g., vaccinations, management of specific disorders).^46^ Finally, the effects of Embrace on participating older adults’ health outcomes, their use of services, and costs must be assessed as well.

## Electronic supplementary material

Below is the link to the electronic supplementary material.

ESM 1 (DOCX 26 kb)

ESM 2 (DOCX 21 kb)
