# Supplementary material for: Integrated Care for Older Adults Improves Perceived Quality of Care: Results of a Randomized Controlled Trial of Embrace
Source: J Gen Intern Med. 2016 Jun 6;32(5):516–23. doi: 10.1007/s11606-016-3742-y (PMC5400746; doi:10.1007/s11606-016-3742-y)
Supplement: Supplementary file 1 — (DOCX 26 kb) [file 11606_2016_3742_MOESM1_ESM.docx]

Online appendix Patient Assessment of Integrated Elderly Care questionnaire

| Patient Assessment of Integrated Elderly Care | | | | | | |
| --- | --- | --- | --- | --- | --- | --- |
| **Over the past six months, when I received care and support for health issues related to ageing or my chronic condition(s), I was…** | *None of the time* | *A little of the time* | *Some of the time* | *Most of the time* | *Always* | *Does not apply* |
| - - - 1. Asked for my ideas and expectations, when we made a care and support plan | □ | □ | □ | □ | □ | □ |
| - - - 1. Given choices about care and support to think about | □ | □ | □ | □ | □ | □ |
| - - - 1. Asked whether I had any problems with my medicines or their (side) effects | □ | □ | □ | □ | □ | □ |
| - - - 1. Asked whether I had any problems with my care and support or what my experiences with either had been | □ | □ | □ | □ | □ | □ |
| - - - 1. Given information on how to stay healthy or improve my health | □ | □ | □ | □ | □ | □ |
| - - - 1. Explained how my own actions or behavior influenced my health | □ | □ | □ | □ | □ | □ |
| - - - 1. Asked which goals I wished to achieve regarding my health | □ | □ | □ | □ | □ | □ |
| - - - 1. Helped to set specific goals in dealing with the consequences of ageing | □ | □ | □ | □ | □ | □ |
| - - - 1. Given a copy of my care and support plan | □ | □ | □ | □ | □ | □ |
| - - - 1. Encouraged to take a course, participate in a group, or undertake activities to help me cope with the consequences of ageing | □ | □ | □ | □ | □ | □ |
| - - - 1. Asked questions, either directly or on a survey, about my lifestyle (e.g., smoking, exercise, diet, etc.) | □ | □ | □ | □ | □ | □ |
| - - - 1. Sure that my healthcare professional had thought about my values, beliefs, and traditions, when they recommended care and support to me | □ | □ | □ | □ | □ | □ |
| - - - 1. Helped to make a care and support plan that I could carry out in my daily life | □ | □ | □ | □ | □ | □ |
| - - - 1. Helped to plan ahead so I could take care of myself in case my health declined or my situation worsened | □ | □ | □ | □ | □ | □ |
| - - - 1. Asked how the consequences of ageing affected my life | □ | □ | □ | □ | □ | □ |
| - - - 1. Contacted after a visit or after participating in a (group) activity to see how things were going | □ | □ | □ | □ | □ | □ |
| - - - 1. Encouraged to attend programs in the community that could help me | □ | □ | □ | □ | □ | □ |
| - - - 1. Referred to a healthcare professional (such as a physical therapist or social worker) or to a (group) activity | □ | □ | □ | □ | □ | □ |
| - - - 1. Explained why a visit to a healthcare professional or participation in an individual or group activity was important for me | □ | □ | □ | □ | □ | □ |
| - - - 1. Asked how my visits to (or by) health-care professionals, or my participation in a (group) activity, were going | □ | □ | □ | □ | □ | □ |

The PAIEC is an adapted version of the PACIC

© Copyright 2004, MacColl Institute for Healthcare Innovation, Group Health Cooperative.

Translated and adapted by R.J.Uittenbroek, S.A.Reijneveld, R.E.Stewart, S.L.Spoorenberg, H.P.Kremer, and K.Wynia

© Copyright 2015 University Medical Center Groningen, Department of Health Sciences

Patient activation and contextual information: (items 1-5,11,12)

Goal setting and problem solving: (items 6-9, 13-15)

Coordination and follow up: (items 10, 16-20)
