# Supplementary material for: Integrated Care for Older Adults Improves Perceived Quality of Care: Results of a Randomized Controlled Trial of Embrace
Source: J Gen Intern Med. 2016 Jun 6;32(5):516–23. doi: 10.1007/s11606-016-3742-y (PMC5400746; doi:10.1007/s11606-016-3742-y)
Supplement: Supplementary file 2 — (DOCX 21 kb) [file 11606_2016_3742_MOESM2_ESM.docx]

**Online supplementary Table S1 Embrace care and support during intervention period, per risk profile^1^**

|  | **Complex care needs** | **Frail** | **Robust** |
| --- | --- | --- | --- |
|  |  |  |  |
| Focus | Health care | Psychosocial | Self-management |
| Elderly Care Team meetings | Monthly meetings, 1-2 hours | | |
| Care and support coordination | Case manager  (district nurse) | Case manager  (social worker) | Elderly Care Team |
| Intensity and duration of individual care and support | 1 to 2 hours home visits,  ±2 per month for  6-12 months | 1 to 2 hours home visits,  ±1 per month for  6-12 months | Does not apply |
| Self-management and prevention program | Once a year full review of medical record, including medications  Community meetings 2/year and newsletters to enhance self-management abilities | | |
| Average percentage of older adults per GP practice^2^ | 20% | 16% | 64% |
| Annual additional cost per older adult^3^ | EUR 1828 | EUR 1463 | EUR 63 |
|  |  |  |  |

^1^ Not included in this table are: costs of annual screening using self-report questionnaires, costs of the Electronic Elderly Records, and the support and training of professionals (staff support 0.1 FTE/year per ECT)

^2^ The average number of older adults 75 years and older per GP practice is 200

^3^ The duration of the intervention at an individual level not taken into account
